# Quantitative investigation on the heterogeneity of deformation fields in sandstone pre-existing cracks during damage evolution

**DOI:** 10.1038/s41598-022-09600-3

**Published:** 2022-04-08

**Authors:** Hongming Cheng, Xiaobin Yang, Yanyu Pei, Yimin Song

**Affiliations:** 1 School of Emergency Management and Safety Engineering, China University of Mining and Technology, Beijing, 100083 China; 2 School of Coal Engineering, Shanxi Datong University, Datong, 037003 China; 3 School of Civil Engineering, North China University of Technology, Beijing, 100144 China

**Keywords:** Solid Earth sciences, Civil engineering

## Abstract

The inherently heterogeneous microstructures of rocks lead to heterogeneity of the deformation distribution within the rock volume. In this study, experiments were conducted on red sandstone specimens with four different pre-existing crack inclinations stressed under uniaxial loading to investigate these features. Acoustic emission and digital image correlation techniques were used to confirm the damage process and obtaining deformation fields, respectively. The results showed that the heterogeneity of the deformation fields in the rock specimens amplifies with increasing stress magnitude, i.e., the displacement field heterogeneity shows two dense bands around the pre-existing crack, and the strain field heterogeneity shows localized regions with a numerical difference (quantified by the normalized standard deviation) and spatial concentration (quantified by the spatial correlation coefficient). The variations in the normalized standard deviation and spatial correlation coefficient were closely related to the damage process. The normalized standard deviation presented four evolution stages: relatively constant low value, steady growth, significant growth, and high rate growth. The spatial correlation coefficient changed from increasing at a relatively constant rate to increasing at a gentle rate and finally increasing rapidly. The evolution rate along with the strain showed two sharp fluctuations. The first could be used as precursor information of the damage. Finally, we confirmed the feasibility of the damage variable obtained from the heterogeneous deformation indicators used to calibrate or form damage evolution laws.

## Introduction

The damage evolution in a rock,which is a heterogeneous natural geological material, proceeds with internal microstructure closure, initiation, propagation, and coalescence form the microscale to the mesoscale and lastly to the macroscale, with a series of physical parameters (sound, electricity, and heat) exhibiting responses^1–3^. Hence, various laboratory-based monitoring techniques have been used to obtain the salient features associated with the damage evolution process, such as the acoustic emission (AE), electromagnetic radiation (EMR), and infrared thermography^4–9^. The responses of the macroscopic stress–strain behaviors and deformation fields (displacement and stain fields) during the damage evolution of rocks are visible, and the deformation measurement in time and space is an essential procedure for analyzing the damage mechanism and fracture process in rocks. The multistage characteristics of a macroscopic stress–strain curve are typically used to identify the damage condition corresponding to the characteristic stresses, which are applied to guide engineering design, construction, and operation stability evaluation; for example, the CI stress (*σ*_ci_) between elastic and plastic transformations is considered for microstructure initiation^10–14^. The deformation field presents heterogeneity, characterized by the formation of localized regions with a high strain (i.e., strain concentration), where a high extensile strain is generated and fractures eventually occur^15–17^.

The heterogeneous deformation fields originate from the heterogeneous microstructure of the rocks^18–20^. Various direct methods, such as X-ray imaging, scanning electron microscopy (SEM), and CT scanning, have been used to study the evolution associated with microstructure^21–26^. However, the aforementioned techniques have the following limitations: (1) The loading needs to be interrupted when analyzing the microstructure evolution; (2) The microstructural analyses are partial because a local region is selected in the experiments; (3) The experimental processes are complicated and expensive, and the large amount of data generated require considerable time for calculations. To overcome these drawbacks, some scholars^19,27,28^ developed numerical techniques to simulate the influence of microscale heterogeneity on the damage process of rocks. However, because of the many hypotheses made, the physical models established though numerical experiments are inconsistent with practical observations. In recent years, the digital image correlation (DIC) technique has been used as a feasible technique to investigate the evolution characteristics of the deformation fields and thus help understand the damage evolution in various types of rocks^29,30^. Because tensile mechanisms dominate the damage initiation and accumulation in rocks, the horizontal strain (*ε*_*xx*_) field or shear strain (*τ*_xy_) field extracted at the characteristic stress is typically selected to study the crack development in intact rocks or rocks with pre-existing cracks though uniaxial compression and tensile tests^31–34^, biaxial tests^35,36^, Brazilian splitting tests^37,38^ and shear tests^39,40^. In the DIC technique, the pixel points on the surface of rocks, typically in the order of thousands, serve as information carriers of the deformation, while only a few pixels with a high strain carry the damage information. Hence, some researchers^17,20,41,42^ calculated the dispersion degree of the deformation fields from a statistical perspective to explain the damage evolution in rocks.

Compared with physical information and microstructure evolution, the acquisition and appearance of heterogeneous deformation fields are more convenient, direct, and comprehensive in identifying the damage condition and precursory information^24,25,32,33^. However, there is scope to quantitatively assess the heterogeneous evolution of the deformation fields along with the damage. Accordingly, in this study, experiments were conducted on red sandstone with four different pre-existing crack inclinations stressed under uniaxial loading, in combination with AE and 2D digital image correlation techniques (2D-DIC) measurements to investigate the heterogeneous deformation fields. Further, two statistical indicators were newly proposed to characterize the numerical difference and spatial gathering in the heterogeneous deformation fields, and the evolutions of these indicators with the loading were explored corresponding to the damage process confirmed by the AE parameters. Additionally, the damage variable was initially quantized from the heterogeneity of the deformation fields, and the stress–strain relationship expressed by the aforementioned damage variable was proven to be feasible by continuum damage mechanics.

## Experimental program

### Specimen preparation

In this study, red sandstone was chosen for the experiment owing to its homogeneous and isotropic nature^43^. X-ray diffraction (XRD) tests were performed to obtain the mineral composition of red sandstone: 63.5% quartz, 14.3% calcite, 12.4% feldspar, 5.5% clay minerals, and 4.3% other minerals. The grain size distribution was in the range of 0.04–0.4 mm. The density of the intact red sandstone specimens was 2.43 g/cm^3^, the uniaxial compressive strength was 87.13 MPa, the tensile strength was 5.52 MPa, and the elastic modulus was 8.31 GPa.

**Figure 1:**
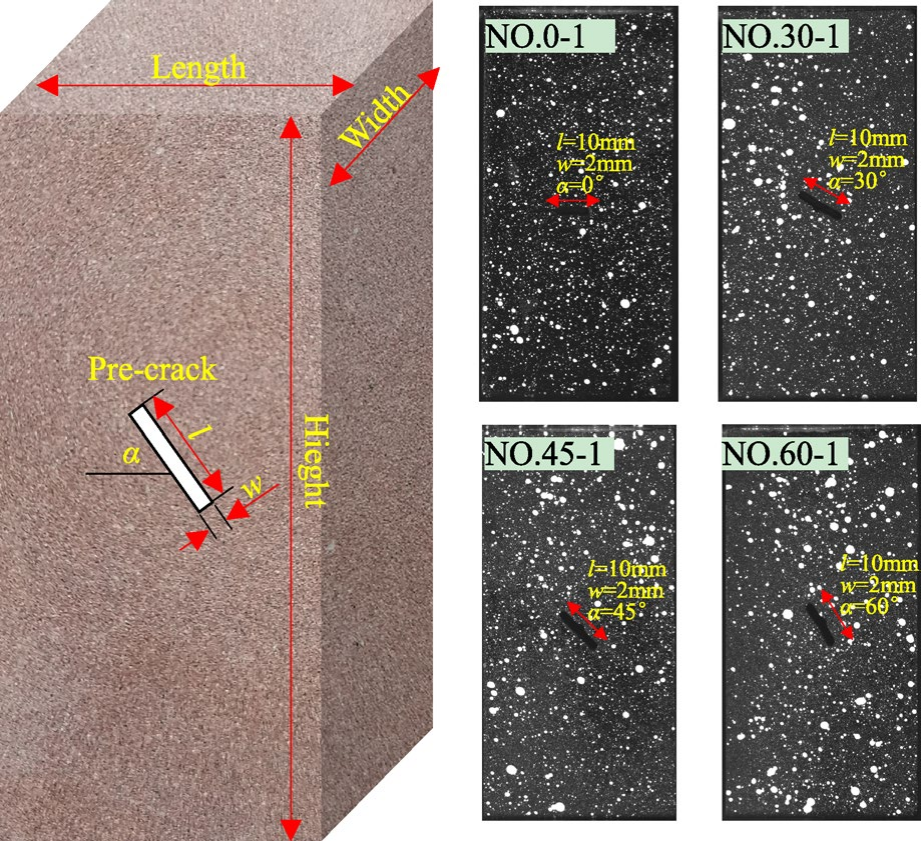
Geometric dimensions and speckle field of some specimens.

All the specimens were carefully cut into standard specimens 50 mm in length, 50 mm in width and 100 mm in height using a circular saw. Subsequently, a crack with 10 mm in length (*l*) and 2 mm in width (*w*) was carefully produced in each specimen using a water-jet cutter, and four different crack inclinations (*α*) were selected: 0°, 30°, 45°, 60°. Finally, a manual speckle field was made as follows: the rock cuttings on the specimen surface were swept, and the specimen surface selected for the deformation monitoring was sprayed with a thin coat of black matte primer. After drying, the white paint was randomly sprayed as speckle. Figure 1 and Table 1 present the geometric dimensions of the specimens with the four different crack inclinations, and the speckle field of some of the specimens to be tested.

**Table 1:** Geometric and mechanical parameters of specimens.

Specimen No	Length (mm)	Width (mm)	Height (mm)	Crack angle (˚)	Peak stress (MPa)	Mean Peak stress (MPa)	Peak stress wreck factor (%)	Elastic modulus (GPa)	Mean elastic modulus (GPa)
0-1	50.22	50.24	100	0	63.50	64.73	74.30%	7.84	7.66
0-2	50.22	50.29	100.05		63.20			7.48	
0-3	50.23	50.27	100.03		67.50			7.65	
30-1	50.01	50.07	99.98	30	65	65.43	75.10%	7.86	7.85
30-2	50.01	50.12	99.95		65.3			7.93	
30-3	50.03	50.08	100		66.00			7.78	
45-1	50.31	50.26	100.02	45	66.20	67.06	76.96%	7.79	7.91
45-2	50.33	50.26	100.07		65.00			7.94	
45-3	50.27	50.28	100.05		70.00			7.99	
60-1	50.29	50.13	100.11	60	73.50	73.63	84.50%	7.84	8.07
60-2	50.31	50.08	100.09		75.00			7.95	
60-3	50.26	50.15	100.12		72.40			8.41	

### Experimental system and procedure

**Figure 2:**
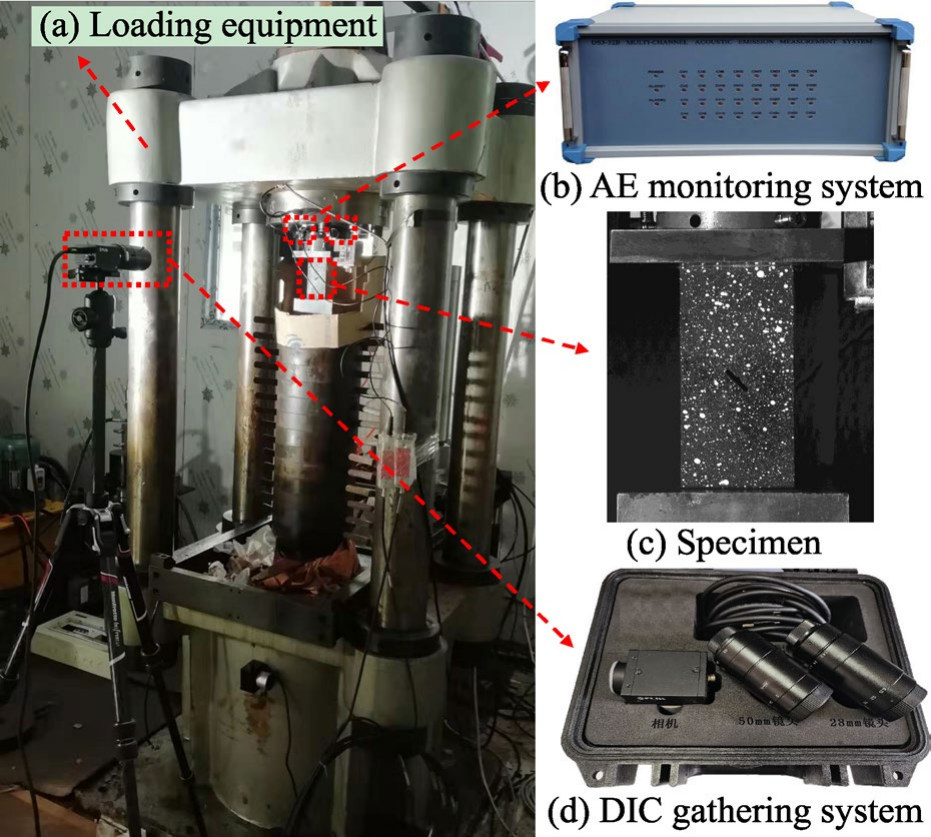
Layout of loading equipment and monitoring systems.

As shown in Fig. 2, the uniaxial compression experimental system consisted of a loading equipment, an AE monitoring system, and a DIC gathering system. The loading equipment was the RLJW-2000 electro-hydraulic servo loading machine with a maximum axial loading capacity of 2000 kN, and the displacement-controlled axial loading was applied at a rate of 0.005 mm/s. The AE monitoring system (manufactured by Vallen Systeme, Germany) recorded the AE parameters (counts, accumulative counts, and energy) though AE sensors during the rock deformation and failure process, two AE sensors were arranged at an angle of 150° on the loading head. The preamplifier gain of the AE monitoring system was set to 40 dB, the threshold value was set to 50 dB, and the sampling rate was 10 MHz. The DIC gathering system comprising a Charge Coupled Device (CCD) industrial camera (Point Grey FL2G-13S2M-C), a lens of 50 mm focal length (Schneider 1001976 2.8/50), and a cold light source was used to gather the speckle images of the specimen surface, with a rate of 10 frames per one second, a graphic resolution of 2048 × 2048 pixels, and an object plane resolution of 0.07 mm/pixel.

To reduce the oversize crack closure stage due to the original pores in the rock specimens, all the specimens were initially loaded with a pre-pressure of 10 kN. The displacement of the press machine was then reset, and the sampling rate and store path of the AE monitoring system and DIC gathering system were adjusted. Finally, the loading equipment, DIC gathering system and AE monitoring system were triggered simultaneously, and the experimental data were collected during the entire loading process and disposed after testing.

## Quantitative indicators of heterogeneous deformation

**Figure 3:**
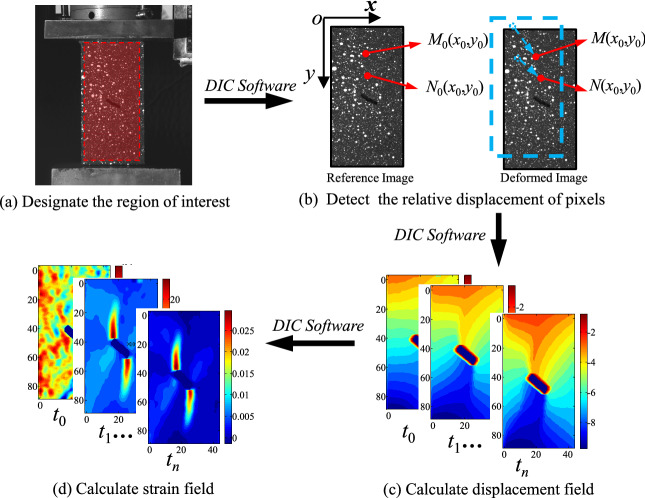
The workflow of DIC.

The heterogeneity of the deformation fields in rock specimens has been widely observed by DIC method in various laboratory tests performed to obtain the key characteristics characteristic during the damage evolution. The DIC method is a common deformation measurement method, with advantages of nondestructive and noncontact measurement. DIC method takes the pixel points in the region of interest (ROI) on the material surface as the information carrier, matches the same pixel points in the reference image and so-called current images through correlation calculation, and obtains the relative displacement before and after deformation, thus realizing the strain measurement in the deformation field of the material surface. Figure 3a–d show the entire DIC method and ROI, and the DIC software acquires from Nanjing Zhongxun Micro Sensing Technology Co., LTD.

Previous studies have shown that the displacement in the dense-ribbon region of the displacement gradient changes rapidly and converges at a position, and the high and low strain differentiation presents two-sided features: spatial gathering and numerical difference. Referring to the idea of statistics, the deformation field information obtained at a certain time (*t*_*k*_) was taken as a sample, a series of *N*_*x*_ × *N*_*y*_ matrices with MATLAB was obtained, including the displacement and strain, representing the temporo-spatial evolution of the deformation behaviors, where *N*_*x*_ and *N*_*y*_ represent the numbers of rows and columns of the matrices, respectively. Let *S*_*ij*_(*t*_*k*_) and [*x*_*j*_, *y*_*i*_](*t*_*k*_) be the matrix element representing the deformation and position, respectively, at time *t*_*k*_ corresponding to the *ij*-th pixel (i and j are the row and column indices, respectively, i = 1, 2,…,*N*_*x*_, j = 1, 2, …, *N*_*y*_).

As shown in Fig. 3b, the plane-coordinate system was set up on the upper left corner of the ROI, with x and y axes representing the width and length of the ROI, respectively. Evidently, each pixel has a definite position on the test surface of the specimen. The rows and columns of the matrices were converted to the coordinate value as follows:

1 $$ \left[ {x_{j} ,y_{i} } \right]\left( {t_{k} } \right) = \left[ {j\frac{W}{PX},i\frac{L}{PY}} \right] $$

where *W* and *L* are the width and length of the ROI, respectively; *PX* and *PY* are the numbers of pixel points on the transverse and longitudinal axes of the ROI.

Based on this, in the heterogeneous deformation fields, the difference in value can be described through statistical indicators: average, variance, standard deviation, and variation coefficient. In space, the spatial correlation coefficient, calculated from the position of the larger deformation point, can be used to quantitatively describe the spatial gathering characteristic.

The average (ADF), variance (VDF), standard deviation (SDDF), and variation coefficient(VCDF) of the heterogeneous deformation fields have been used in previous studies^17,20,41^, which can be expressed as follows:

2 $$ {\text{ADF}}(t_{k} ) = \frac{1}{{N_{x} N_{y} }}\sum\limits_{j = 1}^{{N_{x} }} {\sum\limits_{i = 1}^{{N_{y} }} {S_{ij} \left( {t_{k} } \right)} } $$

3 $$ {\text{VDF}}(t_{k} ) = \frac{1}{{N_{x} N_{y} }}\sum\limits_{j = 1}^{{N_{x} }} {\sum\limits_{i = 1}^{{N_{y} }} {\left( {S_{ij} \left( {t_{k} } \right) - {\text{ADF}}\left( {t_{k} } \right)} \right)}^{2} } $$

4 $$ {\text{SDDF}}(t_{k} ) = \sqrt {\frac{1}{{N_{x} N_{y} }}\sum\limits_{j = 1}^{{N_{x} }} {\sum\limits_{i = 1}^{{N_{y} }} {\left( {S_{ij} \left( {t_{k} } \right) - {\text{ADF}}\left( {t_{k} } \right)} \right)}^{2} } } $$

5 $$ {\text{VCDF}}(t_{k} ) = \frac{1}{{{\text{ADF}}}}\sqrt {\frac{1}{{N_{x} N_{y} }}\sum\limits_{j = 1}^{{N_{x} }} {\sum\limits_{i = 1}^{{N_{y} }} {\left( {S_{ij} \left( {t_{k} } \right) - {\text{ADF}}\left( {t_{k} } \right)} \right)}^{2} } } $$

In this study, the normalized standard deviation (NSDDF) and spatial correlation coefficient (SCCDF) were proposed to characterize the heterogeneity of the deformation fields. The NSDDF is defined as the ratio of the standard deviation of the deformation fields to the maximum standard deviation: the higher the NSDDF, the greater the level of heterogeneity. The NSDDF (*t*_*k*_) can be expressed as follows:

6 $$ {\text{NSDDF}} (t_{k} ) = \frac{{{\text{SDDF}} (t_{k} )}}{{\max ({\text{SDDF}} (t_{k} ))}} $$

The standard deviation of the strain field increases with loading and becomes maximum before the formation of macrocracks^17,20,41^. In other words, the NSDDF ranges from 0 to 1.

In statistics, the Pearson’s correlation coefficient represents the correlation between two random variables. It is expressed as follows:

7 $$ \rho { = }\frac{{E\left[ {\left( {X - E\left( X \right)} \right)\left( {Y - E\left( Y \right)} \right)} \right]}}{{\sqrt {Var\left( X \right)} \sqrt {Var\left( Y \right)} }} $$

The SCCDF could be calculated in a similar form. The specific calculation process is as follow:

1. Selection of deformation parameter. *u*_x_, *u*_y_, *ε*_xx_, *ε*_yy_, and *τ*_xy_ calculated directly by the DIC software have been commonly used in previous studies. Because the tensile mechanisms dominate the processes of damage initiation and accumulation in rocks, it is easy to observe that *ε*_xy_ leads to nucleation for the strain field. Therefore, this study selected the *τ*_xy_ strain field to calculate the SCCDF. The evolution of the *τ*_xy_ strain field was analyzed in Section 3.3.
2. Selection of a wider deformation range. The aforementioned strain field heterogeneity was partitioned dissimilation between the larger deformation points and the smaller ones, and the larger deformation points with a certain range can represent the tensile damage. The top 5%, 10% or 15% larger deformation points can could be selected depending on the situation.
3. Extraction of the coordinate value. *τ*_xy_ was sorted from highest to lowest, and the range was selected. Its coordinate value was extracted using the MATLAB software.
4. Calculation. The SCCDF can be expressed as follows:
  8 $$ {\text{SCCDF}} (t_{k} ) = \frac{{\left| {\sum\limits_{i = 1}^{n} {\left( {x_{i} \left( {t_{k} } \right) - \overline{{x\left( {t_{k} } \right)}} } \right)\left( {y_{i} \left( {t_{k} } \right) - \overline{{y\left( {t_{k} } \right)}} } \right)} } \right|}}{{\sqrt {\sum\limits_{i = 1}^{n} {\left( {x_{i} \left( {t_{k} } \right) - \overline{{x\left( {t_{k} } \right)}} } \right)^{2} } } \sqrt {\sum\limits_{i = 1}^{n} {\left( {y_{i} \left( {t_{k} } \right) - \overline{{y\left( {t_{k} } \right)}} } \right)^{2} } } }} $$
  where
  9 $$ \overline{{x\left( {t_{k} } \right)}} = \frac{1}{n}\sum\limits_{i}^{n} {x_{i} \left( {t_{k} } \right)} $$
  10 $$ \overline{{y\left( {t_{k} } \right)}} = \frac{1}{n}\sum\limits_{i}^{n} {y_{i} \left( {t_{k} } \right)} $$

**Figure 4:**
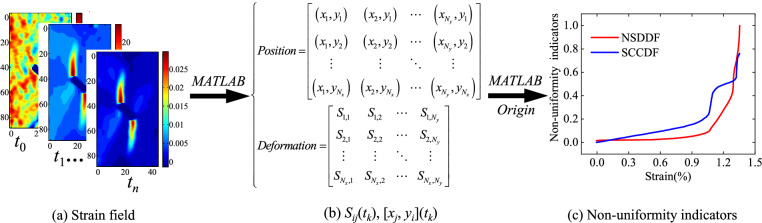
Flowchart of the proposed method for calculating heterogeneous deformation indicators.

A tensile-shear crack initially appeared at the pre-existing crack tips with loading, where larger deformation points gathered, and its correlation coefficient ranged from 0 to 1. Figure 4 shows the process of calculating the heterogeneous deformation indicators.

## Results and discussion

### Strength and damage process

**Figure 5:**
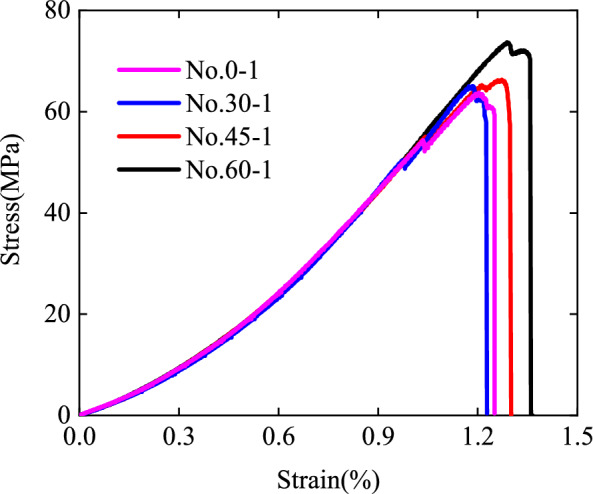
Experimental stress–strain curves: specimen No. 0-1, No. 30-1, No. 45-1, and No. 60-1.

The compressive strength and elastic modulus of the rock specimens with pre-existing cracks gradually increased with increasing crack inclination. As listed in Table 1, the mean compressive strength of the specimen with a 0° pre-existing crack was 64.73 MPa, and the ratio of the mean compressive strength to the intact rock compressive strength (strength wreck factor) was 74.30%. For the 30°, 45°, and 60° pre-existing cracks, the mean compressive strength and wreck factor were 65.43, 67.06, 73.63 MPa and 75.10%, 76.96%, and 84.50%, respectively. The mean elastic modulus values of the sandstone specimens with crack inclination angles of 0°, 30°, 45°, and 60° were 7.66, 7.85, 7.91, and 8.07 GPa, respectively. For the specimen No. 0-1, No. 30-1, No. 45-1, and No. 60-1, Fig. 5 shows the stress–strain curves. Generally, the pre-existing crack specimens with different crack inclination angles experience a primary crack compaction stage, an elastic deformation stage, a new crack development stage, and a post-peak fracture stage. Evidently, the stress dropped in the elastic deformation stage for the specimen No. 0-1, No. 30-1, and No. 45-1, and an unstable development stage existed after the peak stress in all the specimens.

The AE emitted during the propagation and coalescence of microcracks is an effective method to detect the process of rock deformation and damage^4–9^. The AE event reflects the activity of microcracks, and can be quantified using event count rate (ECR) and cumulative event count (CEC). In this study, the ECR and CEC monitored in the tests were normalized and plotted in the same figures as the stress–strain curves. The relationship between the strain and time was linear (due to the displacement-controlled axial loading): *ε* = *at* + *b*; the *a*, *b* parameters are listed in Table 2.

**Table 2:** Fitting parameters between strain and time.

Specimen No	a	b
0-1	0.0025	− 0.0066
30-1	0.0025	− 0.0078
45-1	0.0025	− 0.0063
60-1	0.0025	− 0.0066

**Figure 6:**
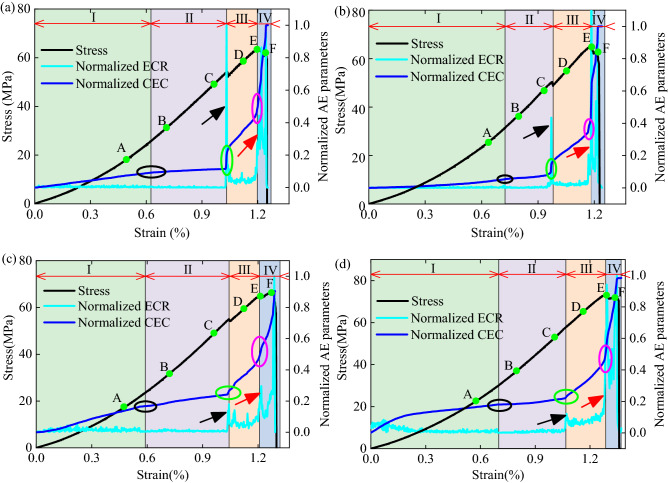
Normalized AE parameters and damage evolution stages for (**a**) Specimen No. 0-1, (**b**) Specimen No. 30-1, (**c**) Specimen No. 45-1, and (**d**) Specimen No. 60-1.

A typical record (specimen No. 0-1, No. 30-1, No. 45-1, and No. 60-1) was chosen for the analysis. As shown in Fig. 6, for the specimens having pre-existing cracks with inclination angles of 0°, 30°, and 45°, the ECR flatly changes before the stress drop, then rapidly increases (see black arrow in Fig. 6a–c) at the stress drop point, thereafter decreases ECR to a level, and maintains its growth with small fluctuations. At the peak stress, the ECR rapidly increases for the second time (see red arrow in Fig. 6a–c), then varies ECR unsteadily after the peak stress. In comparison, in the 60° pre-existing crack specimen, a major surge (see black arrow in Fig. 6d) in the ECR curve can be seen without stress dropping before the peak stress, and the ECR flatly changes and maintains its growth before and after this surge. Another major surge (see red arrow in Fig. 6d) can be observed at the peak stress, after which the ECR fluctuates significantly. For all the pre-existing crack specimens, at the initial loading stage, the CEC maintains slight growth because of the closing of the microcracks and then becomes flat (see black ellipse in Fig. 6). At the ECR surge point (or stress dropping), the CEC exhibits mutation for the first time (see green ellipse in Fig. 6), increases CEC quickly, and appears to show mutation the second time at the peak stress (see purple ellipse in Fig. 6).

According to previous studies on confirming the damage evolution in rock specimens using the AE method^5,44,45^, in this study, the evolution rates of the ECR and CEC were used to confirm the damage evolution in the red sandstone specimens with the four different pre-existing crack inclinations. As shown in Fig. 6, the damage evolution process in all the specimens can be divided into four stages: (I) crack closure stage, (II) linear elastic deformation stage, (III) plastic deformation stage (including crack stable growth and crack unstable growth), and (IV) post-peak stage.

### Temporal and spatial distribution characteristics of heterogeneous deformation fields

**Figure 7:**
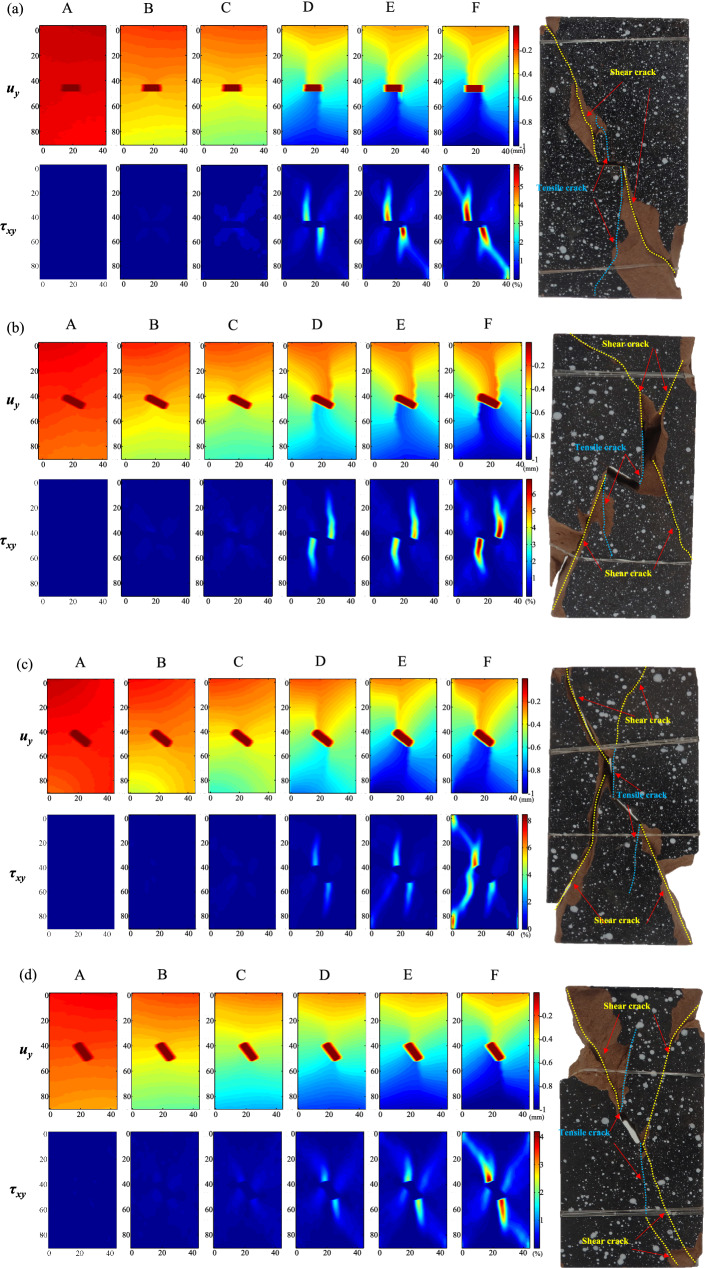
*u*_*y*_ and *τ*_*xy*_ in the surface of rock specimens: (**a**) Specimen No. 0-1, (**b**) Specimen No. 30-1, (**c**) Specimen No. 45-1, and (**d**) Specimen No. 60-1.

The speckle images of the rock surface during the entire loading process were recorded in the test and then processed and analyzed using the DIC software. Further, the displacement and strain fields could be obtained. Six markers (recorded as A, B, C, D, and F) at different stress levels were selected to analyze the temporal-spatial characteristic of the heterogeneous deformation fields. As shown in Fig. 6, the marker A is at the crack closure stage, markers B and C are at the pre- and post- linear elastic deformation stages, respectively, marker D is at the middle plastic deformation stage, marker E is at the peak stress, and marker F is at the post-peak stage. Figure 7a–d show the vertical displacement field (*u*_*y*_) and shear strain field (*τ*_*xy*_) of the specimen No. 0-1, No. 30-1, No. 45-1, and No. 60-1, corresponding to the markers A, B, C, D, E, and F, and the macroscopic fracture morphology (from left to right).

For specimen No. 0-1, No. 30-1, No. 45-1, and No. 60-1, at marker A (i.e., crack closure stage), *u*_*y*_ varied slightly without any evident gradient change; meanwhile, *τ*_*xy*_ did not show any noticeable change. In the linear elastic stage (i.e., markers B and C), due to the initial microcrack closure and lack of new microcrack, *u*_*y*_ changed uniformly with a clear gradient at the bottom and top of the specimens, while at the middle of the specimen, the *u*_*y*_ gradient was cut off by the pre-existing crack, and a serried trend could be observed at both ends of the pre-existing crack, where higher *τ*_*xy*_ gathered. In particular, at marker C, there was an evident partitioned dissimilation of the *τ*_*xy*_ strain field between the larger deformation points and the smaller ones. At marker D, the specimens had undergone plastic deformation with microcrack development, and the *u*_*y*_ displacement field showed an evident heterogeneous change, i.e., two dense bands of the *u*_*y*_ displacement gradient were distributed at both ends of the pre-existing crack. In addition, the direction of the *u*_*y*_ displacement gradient reversed in the dense band and turned into a horizontal change, where the tensile effect appeared initially. Thus, the nonuniformity of the *τ*_*xy*_ strain field, i.e., the partitioned dissimilation between the larger and smaller strain points, was distinct at both ends of the pre-existing crack. With the increase in loading (e.g., the marker E), the dense band of the *u*_*y*_ displacement continually developed, where the heterogeneity of the *τ*_*xy*_ strain field was reinforced. In the post-peak stage, the tiny cracks, covered with paint or not caught by naked eye, had already grown including tensile and shear cracks. As illustrated at marker F in Fig. 7, the deformation degree in the dense band of the *u*_*y*_ displacement field increased beyond the threshold value. Compared with the macroscopic fracture morphology, the tensile cracks appeared in the larger *τ*_*xy*_ gathering zone at both ends of the pre-existing crack (see blue dotted line in Fig. 7), and shear cracks can be found in the zone between the end of the rock specimens and two ends of the pre-existing crack (see yellow dotted line in Fig. 7).

As discussed above, the heterogeneous deformation information could be intuitively obtained from the displacement and strain fields of the rock specimens with the four different pre-existing crack inclinations. In comparison, the damage evolution process could not be quantitively described from the heterogeneous deformation fields.

### Evolution of the heterogeneous deformation indicators

**Figure 8:**
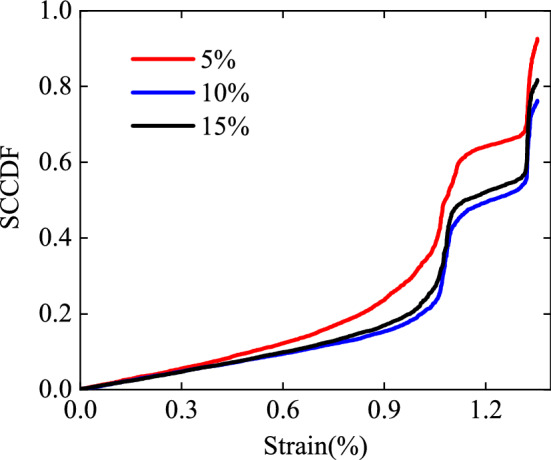
Variation in the SCCDF with loading for the specimen No. 60-1: the top 5%, 10%, and 15% of the *τ*_*xy*_ strain points.

To confirm the suitable range of the *τ*_*xy*_ strain points, taking specimen No. 60-1 as an example, its top 5%, 10%, and 15% of the *τ*_*xy*_ strain points were selected to calculate the SCCDF using the proposed method, and the SCCDF-strain curves were drawn. As shown in Fig. 8, the evolution characteristics of the SCCDF with the loading are approximately same for the three selected ranges. The SCCDF initially increased at a gentle rate, then increased quickly when reaching a certain degree of deformation, subsequently exhibited the first mutation, and decreased after reaching a certain value. Thereafter, the SCCDF again increased at a gentle rate, then increased quickly, and exhibited the second mutation, showing a step-type change overall. However, under the same deformation, the SCCDF decreased with the increasing in the number of selected strain points, while the decreasing extent became slower, e.g., the spatial correlation coefficients of the top 10% and 15% larger strain points are approximately the same. To express the heterogeneity of the strain field more accurately and consider the problem of the calculation amount, the top 10% of the *τ*_*xy*_ strain points were selected for the SCCDF calculation.

**Figure 9:**
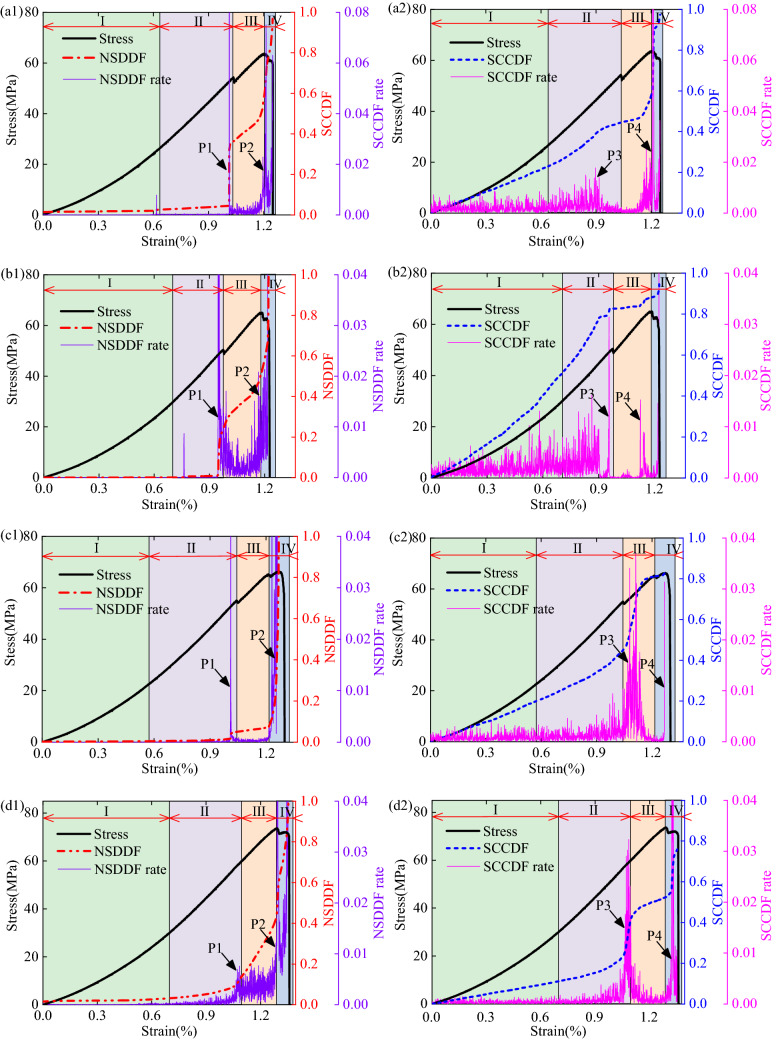
Variations in the NSDDF and SCCDF with loading for the specimen No. 0-1 (**a1**,**a2**), No. 30-1 (**b1**,**b2**), No. 45-1 (**c1**,**c2**), and No. 60-1 (**d1**,**d2**).

Based on the calculation method described in Sect. 3, the NSDDF and SCCDF of the specimen No. 0-1, No. 30-1, No. 45-1, and No. 60-1 were computed. As shown in Fig. 9, although the NSDDF or SCCDF values are not similar to the stress under the same strain levels, almost all the NSDDF–strain curves and the SCCDF–strain curves exhibit the same four stages as the stress–strain curves. The results corresponding to the four stages are discussed as follows:

1. Crack closure stage(I): Due to the low loading and the closure of the initial microcracks, the heterogeneous deformation of the rock specimens were not evident, and the NSDDF value was low and presented a relatively constant trend. However, a part of the larger strain points still gathered around the pre-existing crack, and the SCCDF kept increasing at a relatively constant rate.
2. Linear elastic deformation stage(II): Because of the increasing deformation degree increasing at both ends of the pre-existing crack, the heterogeneous deformation was pronounced. Therefore, the NSDDF value increased, particularly in the later linear elastic deformation stage, increased quickly (see specimen No. 45-1 and No. 60-1 in Figs. 9c1, d1), and even exhibited a mutation (see specimen No. 0-1 and No. 30-1 in Figs. 9a1, b1). The SCCDF still kept increasing at a relatively constant rate, while it increased quickly in the later linear elastic deformation stage and subsequently exhibited a mutation.
3. Plastic deformation stage(III): With microcracks developing in the specimens, the heterogeneous degree of the *τ*_*xy*_ strain field increased, thereby significantly increasing the NSDDF increased drastically. With the *τ*_*xy*_ strain gathering continuously at the end of the pre-existing crack, there was little change in the spatial correlation, and the SCCDF increased at a gentle rate.
4. Post-peak stage(IV): After the peak stress, the macroscopic fracture formed at the larger strain points development area, the heterogeneous degree of the *τ*_*xy*_ strain field increased continuously, and the NSDDF exhibited a second mutation at the peak stress and subsequently kept increasing at a high rate up to the maximum. Also, the spatial correlation of the larger strain points increased, and the SCCDF increased quickly and exhibited a second mutation.

The damage development process of the rock specimens with four different pre-existing crack inclinations could be derived from the heterogeneous deformation. The variations in the NSDDF and SCCDF were closely related to the rock damage; thus, an acceptable agreement was found between the heterogeneous deformation behaviors of these specimens.

As mentioned earlier, the NSDDF and SCCDF expressed the heterogeneity of the *τ*_*xy*_ strain field and showed a specific evolution characteristic at each damage stage. In particular, in the transformation from elasticity to plasticity and at the peak stress, these values increased drastically to varying degrees (similar to the AE parameters). In other words, the evolution rates of the NSDDF and SCCDF were sensitive to key events in the crack development process of the rock specimens. Furthermore, taking the derivative of the normalized standard deviation and the spatial correlation coefficient in the strain field using the finite difference method, the evolution rate (*v*) could be obtained as follows:

11 $$ v_{i} (t_{k} ) = \frac{\partial S}{{\partial t}} \approx \frac{{S_{i + 1} - S_{i - 1} }}{2\Delta t} $$

where *v*_*i*_(*t*_*k*_) is the evolution rate at time *t*_*k*_; *S*_*i-*1_ and *S*_*i*+1_ are the NSDDF or SCCDF value at times *t*_*k-*1_ and *t*_*k*+1_ respectively; Δ*t* is the time interval, Δ*t* = *t*_*k*_-*t*_*k-*1_.

For all the rock specimens (see Figs. 9a1, b1, c1, and d1), *v* of the NSDDF was almost constant at the crack closure stage. While in the linear elastic deformation stage, it fluctuated gently, and fluctuated sharply near the plastic deformation stage (see marker P1 in Figs. 9a1, b1, c1, and d1). Subsequently, the fluctuation in the NSDDF evolution rate changed weakly, and it fluctuated sharply again in the peak stress later (see marker P2 in Figs. 9a1, b1, c1, and d1). The first sharp fluctuation (the marker P1, i.e., the quick increase in the NSDDF) can be used as precursor information of rock fracture, corresponding to the crack initiation stress. As shown in Figs. 9a2, b2, c2, and d2, v of the SCCDF also fluctuates sharply near the plastic deformation stage (see marker P3 in Figs. 9a2, b2, c2, and d2), and fluctuates sharply again in the peak stress later(see marker P4 in Figs. 9a2, b2, c2, and d2), marker P3 could also be used as precursor information of rock fracture.

### Damage quantification from heterogeneous deformation indicators

As discussed previously, the AE signals form the rock specimens increased before sudden failure with the loading, and the strain field showed heterogeneity, indicating the formation of localized regions with numerical difference and spatial gathering. The former could be attributed to the high-frequency elastic waves generated during the development of microcracks in the specimens, whereas the latter was the outward manifestation of the microcracks in the specimen. In other words, there was a similarity between the two in terms of the generating mechanism. Both the AE and heterogeneous deformation field can help describe the damage evolution in the rock specimens. Damage quantification from the AE parameters has been discussed in previous studies^46–48^. Considering the degree and spatial characteristics of the heterogeneous deformation fields, the damage could be quantified from the heterogeneity of the deformation field using the following relationship:

12 $$ D = {\text{NSDDF}} \cdot {\text{SCCDF}} $$

where *D* is the damage variable.

**Figure 10:**
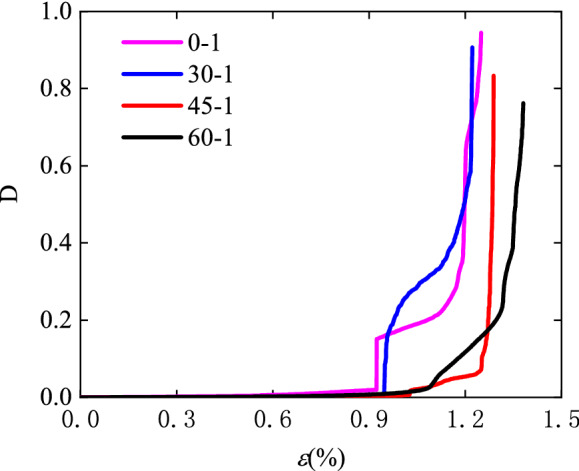
Damage evolution with loading for the specimen No. 0-1, No. 30-1, No. 45-1, and No. 60-1.

Figure 10 shows examples of an individual damage variable from the heterogeneous deformation field for rock specimens with different pre-existing crack inclinations. As the angle increases the damage variable shows a more gradual trend with increasing axial strain. Furthermore, the damage variable can be verified by the theory of continuous damage mechanics.

Using the concept proposed by Lemaitre and Desmorat^49^, the effective stresses during uniaxial compression can be written as:

13 $$ \widetilde{\sigma } = \frac{\sigma }{1 - hD} $$

where σ is the uniaxial stresses, σ̃ is the uniaxial effective stress, and *h* is a microdefects closure parameter that is material-dependent, but most often *h* ≈ 0.2^49^.

**Figure 11:**
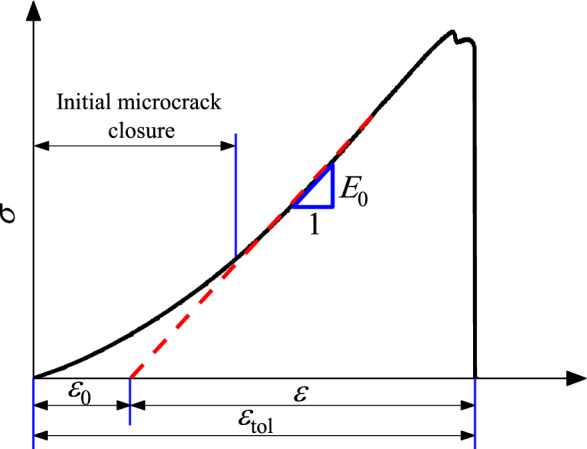
Schematic of *E*_0_, *ε*_0_, *ε*, and *ε*_tol_.

The relationship between the uniaxial stresses and strain can be expressed as:

14 $$ \sigma = \widetilde{\sigma }(1 - hD) = E_{0} \varepsilon (1 - hD) $$

where *E*_0_ is the slope of the linear elastic deformation stage (see Fig. 11 and Table 1), representing the elastic characteristic of the rock framework, and *ε* is the strain of the rock framework.

At a low stress level, the initial microcrack closure controls the rock deformation, while the initial microcrack has only a slight effect on the overall macroscopic behavior of the rocks^50^. Based on this, in Eq. (14), the initial microcrack closure is not considered, where *ε* can be given as follows:

15 $$ \varepsilon = \varepsilon_{tol} - \varepsilon_{0} $$

where *ε*_tol_ is the total strain in the test, and *ε*_0_ is the origin strain of the linear elastic deformation stage (see Fig. 11). For specimen No. 0-1, No. 30-1, No. 45-1, and No. 60-1, the *ε*_0_ values are 0.29, 0.30, 0.33, and 0.32, respectively.

**Figure 12:**
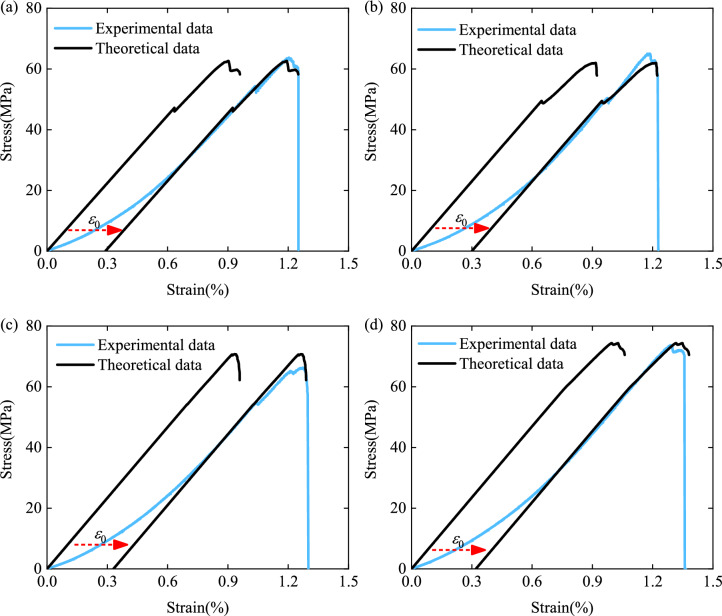
Comparisons between test data and theoretical calculation: (**a**) Specimen No. 0-1, (**b**) Specimen No. 30-1, (**c**) Specimen No. 45-1, and (**d**) Specimen No. 60-1.

Finally, using Eqs. (14) and (15) and the relationship between the damage variable and strain calculated by Eq. (12), the theoretical stress–strain curves form the uniaxial compression tests were obtained. As shown in Fig. 12, moving the theoretical stress–strain curves to the origin of the linear elastic deformation stage in the experimental curves, it can be seen that the theoretical stress–strain curves are in good agreement with the experimental curves after the crack closure stage, i.e., the data obtained from the heterogeneous deformation indicators, used to calibrate or form damage evolution laws, was found to be feasible.

Combining the above observations, it can be concluded that the NSDDF and SCCDF showed evident multistage characteristics and could capture the heterogeneous deformation abnormalities during the damage process by making full use of the full-field deformation information.

## Conclusions

In this study, the crack-induced heterogeneity of the deformation field of red sandstone specimens with different pre-existing crack inclinations was experimentally characterized. The temporal and spatial distribution characteristics of the heterogeneous deformation fields were evaluated in real-time using the DIC method under uniaxial compressive loading. The statistical variations in the strain field of the four rock specimens were also analyzed to achieve the main objective of understanding how it varies in the four damage evolution stages. The following main conclusions can be drawn:

1. The crack-induced heterogeneity of the *u*_*y*_ displacement field showed two dense bands around the two ends of the pre-existing crack, where the *u*_*y*_ displacement gradient reversed, and a tensile effect appeared. In the same regions, the crack-induced heterogeneity of the *τ*_*xy*_ strain field presented numerical difference and spatial concentration, i.e., a high extensile strain was generated and finally fractures occurred.
2. The statistical characteristics quantified by the NSDDF and SCCDF were closely related to the stress–strain state. At the four damage evolution stages, the NSDDF was found to present a relatively constant lower value, a steady increasing trend, a dramatic increasing trend, and increasing with a high rate. Its evolution rate showed two sharp fluctuations near the plastic deformation stage and peak stress; the SCCDF increased at a relatively constant rate, increased at a gentle rate, and increased quickly, also showing two sharp fluctuations. The sharp fluctuations in the NSDDF and SCCDF were reasonably consistent with the CI and peak stress levels of the four rock specimens, and the first sharp fluctuation could be used as precursor information of rock fracture.
3. The damage variable could be quantified by utilizing the heterogeneous deformation indicators, and it occurred more gradually with increasing axial strain and pre-existing crack inclination. The theoretical stress–strain relationship expressed by the forementioned damage variable strongly matched the experimental stress–strain curves of the rock specimens.
